# Tissue Engineering of the Urinary Bladder: Current Concepts and Future Perspectives

**DOI:** 10.1100/tsw.2011.138

**Published:** 2011-07-28

**Authors:** Vladimir Petrovic, Jablan Stankovic, Vladisav Stefanovic

**Affiliations:** University School of Medicine, Nis, Serbia

**Keywords:** urinary bladder, tissue engineering, stem cells

## Abstract

There are many conditions that can affect the normal structure of the urinary bladder wall and lead to the inadequate evacuation of urine or even disable urine excretion. In these cases, the essential task is to restore the function of the urinary bladder, most often through surgical intervention. Some of the disorders, such as bladder acontractility, bladder cancer, and inflammatory disease, represent a great challenge in practice due to the number of complications that can occur after the intervention and due to frequent relapses. The use of tissue engineering strategies that include the use of stem cells and artificially created scaffolds could give solutions for treatment of many disorders of the urinary bladder and transplantation therapies in the future. Although the research in this field is still in its infancy, there are some promising results that raise hope that the tissue engineering approach could offer long-term solutions for many issues in regenerative urology. This review summarizes the current achievements and perspectives in the use of stem cells and tissue engineering techniques in the field of urinary bladder regeneration.

### INTRODUCTION

The increasing need for organ regeneration/replacement imposes the development of new techniques that would be introduced in clinical practice. Tissue engineering and regenerative medicine could contribute to the treatment of many disorders in urology. Although there is an increased number of published studies in animals and a few studies in humans, with very promising results, many of these novel techniques are still being investigated. The field of regenerative medicine is advancing rapidly, so the use of tissue engineering strategies that include the use of stem cells and artificially created scaffolds represent the most probable solution for treatment of many urological disorders and transplantation therapies in the future.

### METHODS IN BLADDER REGENERATION

Today, the most common approach for bladder replacement or repair comprises the use of gastrointestinal segments. However, due to differences in the functions of these two tissues, numerous complications may occur as a result of such a treatment, including hematuria and dysuria syndrome, metabolic abnormalities, infection, urolithiasis, perforation, and even malignancy[1,2,3].

The application of tissue engineering strategies by using the appropriate scaffolds and methods for cell culturing was seen by investigators as a possible solution for these problems. So far, the proposed methods for bladder replacement or repair are tissue expansion for bladder augmentation, use of seromuscular grafts and de-epithelialized bowel segments, as well as the use of tissue engineering strategies[4].

### Augmentation of the Bladder

The treatment of the small-capacity, high-intravesical storage pressure urinary bladder remains a major challenge in urology. Many techniques have been proposed to increase bladder capacity and decrease intravesical pressure. Some of them, such as gastrocystoplasty, colocystoplasty, and ileocystoplasty, are routinely performed in clinical practice, but are related with various complications that may occur postoperatively[1,2,3]. An alternative strategy to decrease intravesical pressure is to use autoaugmentation of the bladder. Various techniques of autoaugmentation include vesicomyotomy, vesicomyectomy, seromuscular enterocystoplasty, and the use of artificial materials, such as autogenous fascia, human dura, pericardium, collagen, and preserved porcine intestinal submucosa, with varying success. The ideal bladder augmentation material has not as yet been identified[5].

Some experiments performed on animals where augmentation cystoplasty was performed with dilated ureteral segments or by causing progressive expansion of native bladder tissue showed promising results. The subsequent urodynamic, histological, and immunocytochemical studies showed that the compliance, structure, and normal phenotypic characteristics of the cells were maintained, while the bladder capacity was significantly increased[6,7].

### Seromuscular Grafts and De-Epithelialized Bowel Segments

The use of seromuscular grafts and de-epithelialized bowel segments has also been the subject of investigation, bearing in mind that this approach avoids the use of intestinal mucosa, and, therefore, keeps the urothelium intact. In that way, the complications associated with the use of the bowel in continuity with the urinary tract could be avoided[8]. Often, seromuscular grafts and de-epithelialized bowel segments are used in combination with enteroplasty augmentation.

González et al. reported that seromuscular colocystoplasty lined with urothelium has proved to be an effective method to augment the bladder in patients who have an artificial urinary sphincter or who undergo simultaneous artificial urinary sphincter implantation[9]. In another study performed by Ardela Díaz et al., “small-bladder” New Zealand rabbits were divided in two groups and subjected to seromuscular colocystoplasty and conventional colocystoplasty. It was reported that the seromuscular colocystoplasty had a poor urodynamic result in improving the capacity and compliance of the urinary bladder compared to conventional colocystoplasty, which showed higher effectivness[10]. In their study with female Wistar rats, Blanco Bruned et al. reported that the technique of seromuscular enterocystoplasty caused high mortality in the rat (63.6%), and that the increase of the vesical volume in groups where demucosalized colocystoplasty and demucosalized colocystoplasty lined by urothelium were performed was small compared to the control group. Only standard colocystoplasty and bladder autoaugmentation produced significant increases on vesical and rupture volume[11].

The benefit of gastric seromuscular flaps was also investigated in a rat model. A gastric seromuscular flap based on an omentum pedicle was transferred to the rat bladder. The results showed that the use of the gastric seromuscular flap in the bladder of a rat resulted in the complete re-epithelialization of the flap and sufficient bladder capacity. However, squamous metaplasia was detected in 30% of the 1-month rats and in 55% of the 4-month rats, as well as the formation of gross calculi in 20% of the 1-month rats and in 34% of the 4-month rats[12]. The use of gastric grafts seeded with urothelial cells or cells from the oral mucosa showed the successful regeneration of the urothelium after transplantation into the urinary bladder of beagle dogs[13,14].

### Tissue Engineering and the Urinary Bladder

Tissue engineering is a multidisciplinary field involving biology, medicine, and engineering, with the main goal to restore, maintain, or enhance the function of tissues and organs[15]. The main requirements for producing an engineered tissue are the presence of adequate stem/progenitor cells, an appropriate extracellular matrix or carrier construct, an adequate blood supply, and the presence of regulatory signals.

There are two approaches in tissue engineering. The acellular approach involves the use of natural or synthetic matrices (scaffolds) to enhance the body's natural ability to repair itself and help in the determination of new tissue growth direction. The cellular approach principle is to use donor cells that are either seeded into the scaffold (cell-seeded scaffold approach) or used alone (the stem cell approach). The source of cells in cell-seeded scaffolds can be from an autologous, allogeneic, or heterologous (xenogeneic) source. The best option is to use autologous cells in order to eliminate the risk of rejection[16].

There are many conditions that can affect the normal structure of the urinary bladder wall and lead to the inadequate evacuation of urine or even disable urine excretion. In these cases, the essential task is to restore the function of the urinary bladder, most often through surgical interventions. Some of the disorders, such as bladder acontractility, bladder cancer, and inflammatory disease, represent a great challenge in practice due to the number of complications that can occur after the intervention and due to frequent relapses. The need to find stable and long-term therapeutic strategies that would resolve these problems incited the increased interest in the field of tissue engineering. The current therapies cannot give the desired results because of their limitations.

Patients with acontractile bladders that can occur as a complication from lower motor neuropathies, severe diabetes, or chronic obstruction often require intermittent catheterizations. Bladder cancer as well as inflammatory diseases of the urinary bladder lead to extensive cell damage, so in these patients, autologous cell harvesting is not possible[17].

Some promising results in the research and use of stem cells and biomaterials in the regeneration of urinary tissues raised a lot of hope, but also a lot of questions that need to be resolved before their widespread use. The current efforts are oriented in two directions. The first is to find the most appropriate stem cells for bladder regeneration and to master their manipulation and control. The second is to design implantable tissue-engineered grafts that display characteristics consistent with the physiology and function of the equivalent healthy native tissue[18]. The role of the grafts is to provide an adequate microenvironment for cell adhesion, proliferation, migration, and differentiation, which would result in the formation of a graft composed of a population of cells resembling the morphology and phenotype of the desired tissue.

### BIOMATERIALS

Scaffolds are constructs that are designed to support cell growth and to provide the accurate tissue architectonic regeneration during the process of healing. Also, the scaffolds can be used as carriers for different growth factors that could enhance the regenerative process. The scaffolds used in bladder tissue regeneration are decellularized natural matrices and synthetic scaffolds.

The decellularized natural matrices can be harvested from autologous, allogeneic, or xenogeneic tissues, and are processed by chemical and mechanical means in order to remove cellular components for eventual implantation[19]. The most used decellularized natural matrices in urology are derived from the bladder tissue or small intestinal submucosa[20]. Recently, it was proposed that the amnion can also serve as a matrix in bladder tissue regeneration[21]. They can be used alone or seeded with cells. However, there are two major problems with the use of decellularized scaffolds: first, tissue below the insertion of the graft must have no underlying pathological changes, and second, the matrix repopulation with the cells does not recapitulate normal developmental, nor a normal tissue repair process[22].

There have been attempts to use many different materials as synthetic scaffolds for urinary bladder regeneration. The most used of these materials in experiments and clinical trials were polyvinyl sponges, Teflon, collagen matrices, Vicryl (PGA, polyglycolic acid) matrices, and silicone. However, these materials failed to successfully regenerate the bladder tissue due to mechanical, structural, functional, or biocompatibility problems[23]. This imposed the need to design new materials that would have adequate structural and biological properties, and would be able to properly direct the cell proliferation and differentiation. Currently, the most commonly used synthetic polymers are poly-α-esters, such as poly(L-lactide) (PLLA), PGA, and poly(lactide-co-glycolic acid) (PLGA). These polymers are biodegradable and exhibit good cell and tissue compatibility[24]. The advantage of using these scaffolds lies in the fact that their physical properties can be closely controlled[25]. Scaffolds can also be used as carriers of different growth factors that can enhance the tissue regeneration and neovascularization. It was observed that incorporation of the basic fibroblast growth factor (bFGF) and vascular endothelial growth factor (VEGF) into an implanted scaffold promoted local angiogenesis[26]. The main obstacle, when incorporating growth factors, remains their controlled release. The promising results in the use of synthetic scaffolds in tissue regeneration are connected with many issues, such as biocompatibility, immunotolerance, rate of degradation, physical properties of the used materials, and effect of degradation products. So far, the ideal biomaterial has not yet been identified[27,28].

### Use of Unseeded Matrices in Bladder Tissue Engineering

The matrices serve as vehicles for partial bladder regeneration, and one of their major advantages is that they do not display relevant antigenicity. The matrices are prepared by mechanically and chemically removing all cellular components from tissue[29,30]. Such acellular substitutes from decellularized scaffolds have been obtained from a variety of tissue sources, including xenogenic and allogeneic small intestinal submucosa and bladder[31,32,33]. The main goal of such an approach would be to obtain differentiated smooth muscle and urothelial cells through the stimulation of the environment to promote cell migration, growth, and differentiation.

A successful regeneration of a mouse bladder by implanting a decellularized bladder matrix scaffold impregnated with fibroblast growth factor was obtained[34]. Bladder regeneration has also been shown to be possible using a small intestinal submucosa matrix, and it was reported that the results were more reliable when the small intestinal submucosa matrix was derived from the distal ileum[35].

In one study, a heterologous and homologous bladder acellular matrix graft and the influence of collagen ratio on the regeneration of function in a dog model was compared. The homologous graft led to more complete regeneration, whereas collagen seemed not to be replaced in the heterologous model, but changed over time. It is possible that the ratio of collagen types seemed to influence smooth muscle regeneration[36].

The normal regeneration of the urothelial layer was observed in many studies where the unseeded grafts were used for cystoplasty, while the muscle layer never fully developed[37,38,39]. However, in some studies, patchy epithelialization of small intestinal submucosa grafts with a mixture of squamoid and transitional cell epithelia was present. One of the findings was the contraction of grafts to 60–70% of their original size, causing little increase in bladder capacity[40,41].

### Use of Cell-Seeded Matrices in Bladder Tissue Engineering

The use of cell-seeded allogeneic bladder matrices has also shown some positive results in bladder regeneration. The study performed on beagle dogs compared the effects of the use of allogeneic acellular matrices obtained from bladder submucosa that were seeded with urothelial and muscle cells obtained from the cystotomy material of five beagle dogs with the unseeded matrices of the same origin. Fluoroscopic and urodynamic tests performed after 2–3 months showed normal bladders and adequate compliance in all cases. Bladders treated with seeded matrices showed a 99% increase in capacity compared to those treated with the acellular matrix, which showed only a 30% increase in capacity. All bladders showed a normal histological structure; however, a larger muscle layer and more nervous fibers were found in the bladders of beagle dogs treated with cell-seeded matrices[42].

Eweida et al. showed positive effects of a urinary bladder matrix (UBM) seeded with keratinocytes in wound healing in the urinary bladder[43]. The UBM is an acellular natural matrix that contains only decellularized basal lamina and lamina propria of the mucosa of the urinary bladder[44]. In the study performed in beagle dogs, keratinocytes seeded on the UBM were transplanted into the urinary bladder of the dogs with full-thickness excision wounds. The positive effects of this treatment on preventing early wound contraction and promoting vasculogenesis were reported[43].

In another study, PGA acellular matrices seeded with urothelial and smooth muscle cells harvested from a suprapubic transmural bladder biopsy were used in beagle dogs after subtotal cystectomy, preserving only the trigone. The control groups consisted of dogs that underwent simple closure of the bladder or bladder reconstruction with a cell-free matrix. The bladders of the dogs treated with PGA acellular matrices seeded with urothelial and smooth muscle cells showed a mean bladder capacity of 95% of the preoperative capacity, compared to the other two groups that showed 20% (treated with simple closure of bladder) and 46% (treated with cell-free matrix). The compliance of the cell-seeded, tissue-engineered bladders was almost no different from preoperative values (106%), compared to the two other groups (10 and 42% total compliance, respectively). The histological and immunohistochemical studies showed a normal three-layer bladder histology in groups of the cell-seeded, tissue-engineered bladders and bladders that underwent simple closure, while there was pronounced fibrosis in bladders treated with the cell-free matrix[42].

A small clinical trial with seven patients suffering from neurogenic bladder was reported. The patients underwent augmentation cystoplasty with acellular matrices seeded with *in vitro*–expanded urothelial and smooth muscle cells. The patients received acellular bladder submucosa matrices or collagen-PGA composite matrices, some with full omental coverage and some without. The use of collagen-PGA cell-seeded scaffolds with omental coverage showed beneficial effects on patients. The most important of these effects were increased compliance and capacity, decreased end-filling pressures, as well as longer dry periods. Biopsies revealed a normal histological structure. The complications related to enterocystoplasty did not appear. This study demonstrated the possibility of using engineered tissue substitutes for partial hollow organ replacement in humans, thus avoiding the need for intestinal substitution[45].

## CELL SOURCES

Stem cells display three important features: they have the ability to self-renew, the ability to differentiate into different cell types, and the ability to form clonal populations. Stem cells can be obtained from early embryonic, fetal, postnatal, or adult sources[46].

- **Embryonic stem cells —** Embryonic stem cells (ESC) can be obtained through aspiration of an embryoblast of a blastocyst or even through isolation of a single cell from this mass. ESC have the ability to differentiate into the cells of all three germ layers and thus represent a very attractive possibility for stem cell research. So far, ESC lines have been derived from mice, nonhuman primates, and humans[47]. However, the clinical use of these cells is limited because they represent an allogeneic resource and therefore have the potential to cause an immune response[46]. Also, these cells showed the ability to form teratomas that contains cells from all three lineages[16]. Currently, the ways to overcome these obstacles are being studied. Another barrier in the use of human ESC are ethical concerns, because creating an ESC line involves destroying a human embryo.

- **Adult stem cells —** Adult stem cells and progenitor stem cells are multipotent cells found in many tissues and organs. The adult mesenchymal stem cells (MSC) from bone marrow are the most investigated and the best understood cells in stem cell biology[48]. So far, bone marrow represents the best source for tissue-derived adult stem cells. MSC were also isolated from adipose tissue, peripheral blood, connective tissues of the dermis, and skeletal muscle. Furthermore, MSC were prepared from other mouse tissues, such as the brain, liver, kidney, lung, thymus, and pancreas[49]. However, the problems associated with stem cell research lies in the fact that some adult stem cells, like the cells isolated from the liver, pancreas, or nervous system, have low proliferative capacity *in vitro* and they are present in a small number in tissues[50]. The main advantage of these cells is that there is no ethical issue concerning their use in research and they do not show the malignant potential when implanted.
- **Stem cells from adipose tissue —** The presence of multipotent stem cells was also reported in stromal elements of adipose tissue[51]. Bearing in mind that adipose tissue is of mesodermal origin, the differentiation potential of these cells could be of great benefit for research and eventual clinical application. They can easily be obtained through lipoaspiration and they represent about 3% of cells present in the lipoaspirate[52]. These cells, which are referred to as adipose-derived stem cells (ADSC), can differentiate toward the osteogenic, adipogenic, myogenic, and chondrogenic lineages when cultured *in vitro*[53,54]. Recently, it has been shown that ADSC also have the ability to differentiate into endothelial cells, smooth muscle cells, and cardiomyocytes when cultured in adequate conditions[55,56]
- **Stem cells from umbilical cord, placenta, and amniotic fluid —** Alternate sources of stem cells are the umbilical cord, amniotic fluid, and placenta. These cells are easily accessible and their use avoids the legal issues. Umbilical cord stem cells are derived from Wharton's jelly, the perivascular mesenchymal area, the umbilical vein, and subendothelial tissues. These cells can be differentiated into adipogenic, osteogenic, chondrogenic, and cardiomyogenic lineages[57]. The amniotic fluid and placenta also contain stem cells that express embryonic and adult stem cell markers[58]. Unlike human ESC, the amniotic fluid stem cells (AFSC) and placental stem cells (PSC) do not form tumors *in vivo*. AFSC can differentiate into the cells of all three germ layers, including cells of adipogenic, osteogenic, myogenic, endothelial, neuronal, and hepatic lineages[58].

### Stem Cells and Bladder Tissue Regeneration

The use of stem cells in the field of regenerative urology is being investigated. In some cases where cystoplasty is not feasible, such as muscle-invasive transitional cell carcinoma or severe persistent hemorrhagic cystitis, where the obtained tissue biopsy could not be used for regenerative cystoplasty, the use of stem cells would be of great benefit to restore the function of the bladder[22].

Bearing in mind that the urinary bladder is mainly composed of two cell types, the main goal of such a therapeutic approach would be to achieve the differentiation of stem or progenitor cells into epithelial urothelial cells and smooth muscle cells, and to obtain the normal histological structure in the newly formed tissue.

An increased number of studies reported the promising results of the use of stem cells in bladder tissue regeneration. Human embryoid body stem cells cocultured with the bladder mesenchyme were successfully differentiated into the urothelium. Even more, the seeding of the coculture of the embryoid body stem cells and the bladder mesenchyme on decellularized xenogenic small intestinal submucosa *in vitro* led to the formation of a composite graft[31,32].

MSC from bone marrow also have the ability to partially transform into smooth muscle cells when treated with cocktails of growth factors and corticosteroids. The same group reported that the combination of stimulation by humoral factors and coculture with primary urothelial cells resulted in a further significant increase of smooth muscle–specific gene expression in the treated MSC, but the cells underwent only partial differentiation[59].

Chung et al. performed augmentation of the rat bladder by introducing porcine small intestinal submucosa seeded with MSC from rat bone marrow. After 3 months, the histological examination showed normal bladder structure in the implanted tissue, with fully differentiated urothelial and smooth muscle cells. Although a three-layered cellular architecture was also observed in control experiments using unseeded small intestinal submucosa, only the stem cell–seeded biohybrid exhibited gene expression levels similar to those of sham-operated animals[60].

Ringdén et al. investigated the effects of allogeneic hematopoietic stem cell transplantation in seven patients with hemorrhagic cystitis. MSC were taken from human donors and given intravenously to the patients. In five patients, the severe hemorrhagic cystitis cleared after MSC infusion and gross hematuria disappeared after a median of 1–14 days. In one advanced case, the perforation healed. In another patient, MSC reduced the need for further transfusions. Although MSC could not be detected, their DNA was found in the host bladder. These results suggest that MSC have the ability to home the damaged bladder tissue and that this therapeutic approach could be of great benefit in the future therapy for tissue toxicity[61]. Tian et al. reported that MSC from bone marrow can be successfully differentiated into smooth muscle cells when seeded on a highly porous PLLA scaffold and treated with platelet-derived growth factor BB (PDGF-BB) plus tumor growth factor β1 (TGF-β1)[62].

ADSC have been able to increase smooth muscle gene expression in response to dexamethasone and hydrocortisone[63]. Also, the successful regeneration of smooth muscle cells of the urinary bladder in rats was achieved through the use of ADSC and 85:15 PLGA scaffold[52].

In another study, it was reported that endothelial progenitor cells have characteristics applicable for bladder tissue regeneration because they express similar contractile and vascular markers as bladder wall elements[64]. There are suggestions that epithelial and dermal multipotent stem cells within hair follicles could be of benefit for tissue engineering of the urinary bladder. Drewa et al. investigated the suitability for cystoplasty augmentation in rats of *in vivo* implanted acellular bladder matrices previously seeded with hair follicle stem cells and of matrices implanted without the cells. The results showed that bladder capacity was similar in both groups, but the shape was regular and characteristically oval only in bladders grafted with cell-seeded acellular matrices. Muscle layers were extremely thin in the cases of acellular grafts and thicker in bladders reconstructed with cell-seeded grafts. The urothelium regenerated in all animals[65].

Recently, a subpopulation of cells isolated from urine was demonstrated to have progenitor cell characteristics and the potential to differentiate into urothelial, smooth muscle, endothelial, and interstitial cells. After a serial culture, these cells maintained normal karyotype. Further research is needed to establish whether these cells could be used in tissue engineering of the urinary tract[66].

### PERSPECTIVES

The main goal of bladder regeneration is to obtain a fully functional bladder with accurate histological structure. The methods used in today's clinical practice in urology, although with positive results, are connected with the occurrence of many complications. Tissue engineering methods, which comprise the use of matrices, scaffolds, and stem cells, raise the possibility of creating more effective treatments that could be of greater benefit for the patients and that could minimize the negative effects of current procedures.

Our understanding of the underlying mechanisms involved in tissue regeneration is still insufficient. Although the results are promising, most of our knowledge is based on experimental studies, without verification in clinical work. The lack of suitable *in vitro* differentiation protocols for adult stem cells has led to strategies that apply native stem cells, predifferentiated stem cells, or committed precursors for transplantation. The use of ESC remains controversial due to their possible tumorigenicity and ethical dilemmas about their use. On the contrary, the use of adult stem cells from autologous tissue has demonstrated sufficient clinical utility for *in vitro* expansion and tissue regeneration. So far, the use of stem cells has shown only partial results in the regeneration of the urinary bladder. The most examined stem cells are MSC, but the other multipotent stem cells isolated from adipose tissue, amniotic fluid, hair follicles, and urine were shown to have the ability to transform into different types of urinary bladder cells. What we need is to make further effort to master the differentiation of these cells and to maintain control over them after transplantation. The combination of stem cells with synthetic or natural scaffolds, with the use of appropriate growth factors, should be further examined in order to regenerate the fully functional urinary bladder. Also, advances in technical sciences should offer us new and improved methods for scaffold preparation, in terms of their strength and functional characteristics, as well as their biological properties.

The prerogatives for the use of tissue engineering in the treatment of disorders of the urinary bladder require the mastering of the techniques of cell harvesting, culturing, and expansion of cells, as well as scaffold design[67].

### ACKNOWLEDGMENTS

This work was supported by a grant, No 175092, from the Ministry of Education and Science of Serbia.
